# Effectiveness of Antibiotic Cement-Coated Rods in the Management of Chronic Post-traumatic Osteomyelitis

**DOI:** 10.7759/cureus.96685

**Published:** 2025-11-12

**Authors:** Muhammad Naqqash, Mubbshir Khan, Haroon Yousaf, Atizaz Ali Jan, Uday Mahajan, Maria Ahmad

**Affiliations:** 1 Trauma and Orthopaedics, University Hospitals Birmingham NHS Foundation Trust, Birmingham, GBR; 2 Trauma and Orthopaedics, Hayatabad Medical Complex, Peshawar, PAK; 3 Trauma and Orthopaedics, Khyber Teaching Hospital, Peshawar, PAK; 4 Trauma and Orthopaedics, Queen Elizabeth Hospital Birmingham, Birmingham, GBR; 5 Trauma and Orthopaedics, Mardan Medical Complex, Mardan, PAK; 6 Trauma and Orthopaedics, University Hospital Crosshouse, Crosshouse, GBR; 7 Trauma and Orthopaedics, Saidu Group of Teaching Hospitals, Swat, PAK; 8 Diagnostic Radiology, Mardan Medical Complex, Mardan, PAK

**Keywords:** antibiotic cement-coated rods, bone healing, chronic osteomyelitis, infection control, intramedullary nailing

## Abstract

Background: Chronic post-traumatic osteomyelitis (CPTO) remains a challenging orthopedic condition characterized by persistent infection, bone destruction, and delayed healing.

Objective: This study aimed to evaluate the short-term effectiveness of locally fabricated antibiotic cement-coated rods in infection control and bone healing among patients with CPTO of the femur and tibia.

Methodology: This descriptive study was conducted at the Department of Orthopedics, Hayatabad Medical Complex, Peshawar, Pakistan, from September 9, 2022, to March 9, 2023. A total of 126 patients aged 18-60 years with CPTO were included using consecutive non-probability sampling. Patients with non-union, segmental bone defects, or Cierny-Mader type IV osteomyelitis were excluded. All patients underwent standardized surgical debridement followed by the insertion of antibiotic cement-coated intramedullary rods prepared intraoperatively with vancomycin (2 g) and gentamicin (0.5 g) per 40 g of cement. Postoperative antibiotics were adjusted according to intraoperative culture and sensitivity results. Clinical and radiological assessments were performed at three and six weeks and at three months. Data were analyzed using IBM SPSS Statistics software, version 24 (IBM Corp., Armonk, NY).

Results: Of 126 patients, 77 (61.11%) were male and 49 (38.89%) female, with a mean age of 40.07 ± 11.44 years. The femur was involved in 96 patients (76.19%), and the tibia in 30 patients (23.81%). Treatment was effective in 108 patients (85.71%), while 18 patients (14.29%) experienced persistence or recurrence of infection. Pain relief improved from 78 patients (61.90%) at three weeks to 110 (87.30%) at three months. Sinus discharge resolved in 112 patients (88.89%) at three months. Radiologically, callus formation increased from 52 patients (41.27%) at three weeks to 101 (80.16%) at three months, and the absence of sequestrum improved from 90 (71.43%) to 113 (89.68%).

Conclusion: Antibiotic cement-coated rods demonstrate high short-term effectiveness in infection control and bone healing in CPTO. However, further multicenter studies with longer follow-up and standardized microbiological reporting are warranted to confirm long-term efficacy and reproducibility.

## Introduction

Persistent infection, repeated drainage, bone deterioration, and poor healing that are commonly associated with chronic osteomyelitis continue to make it one of the most difficult orthopedic disorders to treat [1,2]. The treatment of this illness continues to be challenging owing to the production of biofilm, limited antibiotic penetration into infected bone tissue, and high recurrence rates [3]. Furthermore, despite breakthroughs in surgical procedures and antimicrobial therapy, this condition continues to be difficult to treat. The chronic nature of the illness not only makes it difficult for limbs to function properly, but it also puts patients at risk for protracted impairment, multiple surgical procedures, and even amputation in extreme situations [4].

Traditional treatment methods, such as systemic antibiotic medication and surgical debridement, are usually insufficient in attaining the goal of eradicating an infection in a way that is long-lasting [5]. It is common for systemic antibiotics to fail to maintain therapeutic concentrations inside the diseased bone, and severe debridement may further weaken the bone's structural integrity [6]. The development of local antibiotic delivery systems has provided a potential alternative. These systems attempt to overcome the limits of systemic treatment by reaching high local concentrations of antimicrobial drugs directly at the infection site while simultaneously limiting the toxicity of the antibiotics to the organs and tissues throughout the body [7].

When it comes to the many different methods of local distribution, antibiotic-impregnated cement has garnered a large amount of clinical interest [8,9]. It is possible to successfully handle the combined difficulties of infection management and bone stabilization by using cement-coated intramedullary rods, which are intended to offer both structural stability and prolonged local release of antibiotics [10]. These devices lower the bacterial burden, limit the formation of biofilm, and produce an environment that is more conducive to bone healing and reconstructive treatments [11]. They do this by maintaining high local antibiotic concentrations. Additionally, cement-coated rods are reasonably simple to produce and adapt intraoperatively, making them a solution that is both practical and cost-effective in instances when there are limited resources available in healthcare settings [12].

Antibiotic cement-coated rods have been shown to have positive results in a number of investigations, including the elimination of infections, the enhancement of limb function, and the decrease of recurrences. On the other hand, due to the fact that patient demographics, infection characteristics, and treatment regimens might vary greatly from one another, more clinical study is required in order to determine whether or not this method is beneficial in varied situations.

The objective of this study was to determine the effectiveness of antibiotic cement-coated rods in patients with chronic osteomyelitis.

## Materials and methods

Study design and setting

This descriptive study was conducted at the Department of Orthopedics, Hayatabad Medical Complex, Peshawar, Pakistan, over a six-month period from September 9, 2022, to March 9, 2023.

Inclusion and exclusion criteria

Both male and female patients aged 18 to 60 years who had been diagnosed with chronic post-traumatic osteomyelitis (CPTO) of the femur or tibia, had a disease duration of more than three months, and were scheduled for intramedullary nailing with antibiotic cement-coated rods were included. Eligibility was limited to individuals with American Society of Anesthesiologists (ASA) physical status I or II [13].

Patients were excluded if they had segmental bone defects resulting from debridement or trauma (Cierny-Mader type IV osteomyelitis), non-union at the time of planned nailing, active systemic infection or immunocompromised state, were non-consenting, or were lost to follow-up during the study period.

Sample size

A sample size of 126 patients was calculated using the WHO sample size calculator [14], based on the formula: $n = \frac{Z^2 \times p \times (1 - p)}{d^2}$ where n = required sample size; Z = 1.96 (for 95% confidence level); p = expected proportion of treatment efficacy (0.80); and d = margin of error (0.07). 

By substituting these values, $n = \frac{(1.96)^2 \times 0.80 \times (1 - 0.80)}{(0.07)^2} = 125.4$. 

Thus, a total of 126 patients were included in the study. Participants were recruited through consecutive non-probability sampling.

Data collection

A structured proforma, developed by the orthopedic surgical team, was used to systematically record demographic and clinical data, including age, gender, duration of symptoms, comorbidities (such as diabetes mellitus and hypertension), smoking status, and residential background (Appendix A). All eligible patients were enrolled after obtaining written informed consent.

Preoperative evaluation included a detailed medical history, focused musculoskeletal examination, and baseline laboratory investigations (complete blood count, erythrocyte sedimentation rate, C-reactive protein, and radiographs of the affected limb). These assessments ensured accurate diagnosis, infection staging, and surgical planning.

Surgical procedure and postoperative management

All surgical procedures were performed by orthopedic consultants with a minimum of two years of operative experience under spinal or general anesthesia, depending on patient fitness and surgical requirements. Any previous implants were removed, and a cortical window was created at the site of the sequestrum. Meticulous debridement was performed to excise all necrotic and infected tissue until healthy, bleeding bone was encountered. After thorough debridement, multiple bone specimens were collected for histopathological examination and microbiological culture with sensitivity testing. The medullary canal was reamed and irrigated with approximately six liters of normal saline to ensure optimal decontamination.

Antibiotic Cement-Coated Rod Preparation

Antibiotic-coated intramedullary rods were prepared intraoperatively under sterile conditions using Simplex P bone cement (40 g) mixed with vancomycin 2 g and gentamicin 0.5 g. The mixture was hand-mixed to achieve uniform antibiotic dispersion and molded over Ender or interlocking nails, depending on canal diameter. A cement coating thickness of approximately 2-3 mm was maintained for optimal fit and elution characteristics. After partial polymerization, the rods were shaped for smooth insertion and allowed to harden before implantation.

Postoperative Care and Follow-Up

All patients received empirical intravenous ceftriaxone (1 g twice daily) and amikacin (500 mg twice daily) for the first five postoperative days, followed by oral levofloxacin (500 mg once daily) for four weeks. The antibiotic regimen was subsequently adjusted based on culture and sensitivity results. Wound care and physiotherapy were initiated as tolerated, with weight-bearing restricted until early radiological signs of healing were evident.

Outcome Evaluation

Clinical and radiological follow-ups were conducted at three weeks, six weeks, and three months postoperatively. Treatment success was defined as complete resolution of infection, evidenced by absence of pain, sinus discharge, and local inflammation, along with radiological findings of callus formation and absence of sequestrum or lytic changes.

Statistical analysis

Data were analyzed using IBM SPSS Statistics software, version 24 (IBM Corp., Armonk, NY, USA). Continuous variables, such as age and duration of illness, were first tested for normality using the Shapiro-Wilk test. Normally distributed variables were summarized as mean ± standard deviation (SD), whereas non-normally distributed variables were expressed as median and interquartile range (IQR). In this study, the duration of illness demonstrated a skewed distribution and was therefore presented as median (IQR) to ensure accurate representation of the data.

Categorical variables, including gender, smoking status, comorbidities, affected bone, and treatment efficacy, were described as frequencies and percentages. To control for potential confounding factors, stratification was performed based on key demographic and clinical variables. Post-stratification analyses were conducted using the chi-square test to assess associations between categorical variables. A p-value < 0.05 was considered statistically significant.

Ethical approval

Ethical approval was obtained from the Institutional Ethical Committee of Hayatabad Medical Complex, Peshawar (approval number: 796/HEC/B&PSC/2022). Written informed consent was obtained from all participants, with assurance of confidentiality and voluntary participation.

## Results

The mean age of patients was 40.07 ± 11.44 years, with 62 patients (49.21%) in the 36-50-year age group. The duration of illness was non-normally distributed and therefore presented as median (IQR). Seventy-seven patients (61.11%) were male, and the femur was the most commonly affected bone in 96 patients (76.19%), as shown in Table 1. Comorbidities included diabetes mellitus in 39 patients (30.95%) and hypertension in 53 patients (42.06%). A majority were non-smokers (85 patients, 67.46%) and urban residents (72 patients, 57.14%).

**Table 1:** Baseline characteristics of study participants Continuous variables are presented as mean ± SD or median (IQR), depending on data distribution.

Variables	Categories	Number of Patients (n; %)
Age (years)	Mean ± SD	40.07 ± 11.44
	18–35	41 (32.54)
	36–50	62 (49.21)
	51–60	23 (18.25)
Duration of complaints (days)	Median (IQR)	9 (7–12)
Gender	Male	77 (61.11)
	Female	49 (38.89)
Bone involved	Femur	96 (76.19)
	Tibia	30 (23.81)
History of diabetes	Yes	39 (30.95)
	No	87 (69.05)
History of hypertension	Yes	53 (42.06)
	No	73 (57.94)
Smoking status	Smoker	22 (17.46)
	Non-smoker	85 (67.46)
	Ex-smoker	19 (15.08)
Residential status	Urban	72 (57.14)
	Rural	54 (42.86)

**Figure 1:**
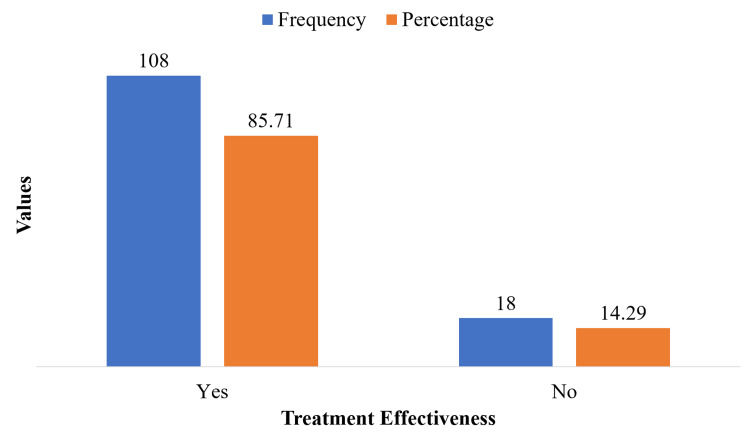
Treatment effectiveness of antibiotic cement-coated rods in chronic osteomyelitis

Out of 126 patients, treatment with antibiotic cement-coated rods was effective in 108 patients (85.71%), while 18 patients (14.29%) experienced recurrence or persistence of infection, reflecting a high overall success rate (Figure 1).

Stratification analysis showed no significant association between treatment effectiveness and age (p = 0.45), gender (p = 0.29), bone involved (p = 0.17), diabetes (p = 0.75), hypertension (p = 0.82), smoking status (p = 0.86), residential status (p = 0.88), or duration of complaints (p = 1.00), as shown in Table 2. For example, treatment was effective in 55 of 77 males (71.43%) vs. 53 of 49 females (70.27%), and in 30 of 39 diabetics (76.92%) vs. 78 of 87 non-diabetics (89.66%), with differences not statistically significant.

**Table 2:** Stratification of treatment effectiveness with different variables (n = 126) Chi-square test (χ²) was applied. A p-value ≤ 0.05 was considered statistically significant.

Variable	Categories	Yes; n (%)	No; n (%)	χ² (df)	p-value
Age (years)	18–35	33 (30.56)	8 (44.44)	1.60 (2)	0.45
	36–50	54 (50.00)	8 (44.44)		
	51–60	21 (19.44)	2 (11.11)		
Gender	Male	68 (62.96)	9 (50.00)	1.11 (1)	0.29
	Female	40 (37.04)	9 (50.00)		
Bone involved	Femur	80 (74.07)	16 (88.89)	1.85 (1)	0.17
	Tibia	28 (25.93)	2 (11.11)		
Diabetes	Yes	34 (31.48)	5 (27.78)	0.10 (1)	0.75
	No	74 (68.52)	13 (72.22)		
Hypertension	Yes	45 (41.67)	8 (44.44)	0.05 (1)	0.82
	No	63 (58.33)	10 (55.56)		
Smoking status	Smoker	19 (17.59)	3 (16.67)	0.30 (2)	0.86
	Non-smoker	72 (66.67)	13 (72.22)		
	Ex-smoker	17 (15.74)	2 (11.11)		
Residential status	Urban	62 (57.41)	10 (55.56)	0.02 (1)	0.88
	Rural	46 (42.59)	8 (44.44)		
Duration of complaints (days)	5–10	69 (63.89)	12 (66.67)	0.00 (1)	1
	>10	39 (36.11)	6 (33.33)		

Clinical and radiological follow-up showed progressive improvements (Table 3). Pain relief was achieved in 78 patients (61.90%) at three weeks, 97 patients (76.98%) at six weeks, and 110 patients (87.30%) at three months (p < 0.001). Absence of sinus discharge improved from 82 patients (65.08%) at three weeks to 112 patients (88.89%) at three months (p < 0.001). Wound healing was observed in 75 patients (59.52%) at three weeks, 93 patients (73.81%) at six weeks, and 106 patients (84.13%) at three months (p < 0.001). Recurrence of infection was seen in 12 patients (9.52%) at three weeks, 14 patients (11.11%) at six weeks, and 18 patients (14.29%) at three months, with late recurrence contributing to the final number (p = 0.108). Radiologically, callus formation increased from 52 patients (41.27%) at three weeks to 101 patients (80.16%) at three months (p < 0.001). Absence of sequestrum improved from 90 patients (71.43%) at three weeks to 113 patients (89.68%) at three months (p < 0.001). Adequate canal clearance was observed in 94 patients (74.60%) at three weeks, 103 patients (81.75%) at six weeks, and 109 patients (86.51%) at three months, showing borderline significance (p = 0.053).

**Table 3:** Clinical and radiological outcomes at follow-up (n = 126) Chi-square test (χ²) was applied. A p-value ≤ 0.05 was considered statistically significant.

Outcomes		3 weeks; n (%)	6 weeks; n (%)	3 months; n (%)	χ²	p-value
Clinical outcomes	Pain relief	78 (61.90)	97 (76.98)	110 (87.30)	22.16	<0.001
	Absence of sinus discharge	82 (65.08)	98 (77.78)	112 (88.89)	20.35	<0.001
	Wound healing	75 (59.52)	93 (73.81)	106 (84.13)	19.29	<0.001
	Recurrence of infection	12 (9.52)	14 (11.11)	18 (14.29)	4.45	0.108
Radiological outcomes	Callus formation/bone healing	52 (41.27)	76 (60.32)	101 (80.16)	39.90	<0.001
	Absence of sequestrum	90 (71.43)	104 (82.54)	113 (89.68)	13.98	<0.001
	Adequate canal clearance	94 (74.60)	103 (81.75)	109 (86.51)	5.87	0.053

## Discussion

Chronic osteomyelitis is still a difficult condition to treat in orthopedics, often requiring creative methods to effectively manage infections and promote bone repair. The purpose of this research was to assess the effectiveness of antibiotic cement-coated rods in treating femoral and tibial CPTO. With an 85.71% treatment success rate, we showed that antibiotic cement-coated rods were useful in treating CPTO. This is consistent with earlier research that found that 38 patients with persistent osteomyelitis treated with cement rod spacers and targeted antibiotics had an overall favorable clinical result of 84.2% [15]. Similarly, 30 patients treated with antibiotic cement-coated pins had a 96.66% success rate, according to research by Garabano et al. (2021) [16].

At three months, our cohort's infection recurrence rate was 14.29%, which is similar to the 16.9% recurrence rate seen in a study of 314 limb osteomyelitis patients treated with cement spacers loaded with antibiotics for the ultimate therapy of bone defects [17]. Nonetheless, our research did not discover any noteworthy correlations between recurrence and factors like age, gender, bone involved, or comorbidities, indicating that the efficacy of the therapy was uniform across all patient subgroups.

Our study's clinical results demonstrated significant progress over time. At three months, 87.30% of patients reported some pain reduction, and 84.13% said that their wounds had healed. These results are in line with prior research that found that 40 patients treated with antibiotic-impregnated bone cement rods for postoperative infections after femoral shaft fractures had a 100% infection control rate and full osseous union at 12 months of follow-up [18].

Our study's radiological results show significant improvements in callus development and sequestrum absence over time. At three months, 89.68% of patients had no sequestrum, and 80.16% of patients had callus development, demonstrating successful bone repair. These outcomes are similar to those of another trial that found no evidence of osteomyelitis recurrence in patients treated with cement spacers impregnated with antibiotics during a mean follow-up of two years [9].

Our research contributes to the growing body of data demonstrating the effectiveness of cement-coated rods coated with antibiotics in the treatment of persistent osteomyelitis. This method not only improves infection management but also promotes bone regeneration and lowers the chance of recurrence by administering high local concentrations of antibiotics straight to the infection site.

Study strengths and limitations

This study demonstrated several strengths, including standardized surgical procedures, a clearly defined patient cohort, and structured follow-up at multiple intervals, which enabled a detailed assessment of both clinical and radiological outcomes. The inclusion of a relatively large sample size (n = 126) and diverse demographic characteristics supports the generalizability of the findings.

Several limitations must be acknowledged. The single-center design, short follow-up duration (three months), and absence of a control group restrict the ability to draw causal inferences or evaluate long-term treatment effectiveness. Moreover, the study did not employ standardized quantitative outcome metrics for infection resolution or bone healing, which could have enhanced objectivity and comparability. The incomplete documentation of postoperative antibiotic regimens and the limited microbiological characterization of pathogens represent additional analytical constraints. Variations in osteomyelitis severity, previous surgical interventions, and comorbid factors may also have influenced the observed outcomes.

Accordingly, while the interpretation of results aligns with the descriptive data, showing consistent short-term clinical and radiological improvement, it should be viewed as observational and preliminary, not confirmatory. Causal or long-term effectiveness claims cannot be substantiated based on the current design. Future studies should incorporate multicenter randomized controlled designs, standardized outcome measures, detailed microbiological profiling, and extended follow-up periods to validate and expand upon these findings.

## Conclusions

According to the study’s findings, antibiotic cement-coated rods provide high short-term effectiveness in controlling infection, reducing pain, promoting wound healing, and enhancing radiographic bone regeneration in patients with CPTO. The combination of mechanical stability and sustained local antibiotic delivery offers a practical and reliable treatment approach for complex bone infections. However, given the descriptive design, limited follow-up duration, and absence of detailed microbiological profiling, these results should be interpreted cautiously. Future multicenter studies with standardized outcome measures and longer follow-up are warranted to validate the long-term efficacy and reproducibility of this technique.

## Appendices

Appendix A

**Table 4:** A structured proforma, developed by the orthopedic surgical team, was used to systematically record demographic and clinical data, including age, gender, duration of symptoms, comorbidities (such as diabetes mellitus and hypertension), smoking status, and residential background.

Section	Variable	Response Options / Format
Patient Demographics	Study ID	__________
	Hospital Registration No.	__________
	Age (years)	__________
	Gender	☐ Male ☐ Female
	Occupation	__________
	Residential Background	☐ Urban ☐ Rural
	Socioeconomic Status	☐ Low ☐ Middle ☐ High
Clinical History	Mechanism of Initial Injury	☐ Road Traffic Accident ☐ Fall ☐ Gunshot ☐ Other: __________
	Time Since Injury (months)	__________
	Duration of Symptoms (months)	__________
	Previous Surgeries at Affected Site	☐ Yes ☐ No (If yes, specify type and number): __________
	Previous Antibiotic Use	☐ Yes ☐ No (If yes, specify duration and type): __________
	Sinus Tract Present	☐ Yes ☐ No
	Active Discharge	☐ Serous ☐ Purulent ☐ None
	Pain Severity (VAS 0–10)	__________
Comorbidities and Risk Factors	Diabetes Mellitus	☐ Yes ☐ No
	Hypertension	☐ Yes ☐ No
	Smoking Status	☐ Smoker ☐ Non-smoker ☐ Ex-smoker
	Alcohol Use	☐ Yes ☐ No
	Nutritional Status / BMI	__________
	Immunosuppressive Conditions	☐ Yes ☐ No (specify): __________
Local Examination	Site of Osteomyelitis	☐ Femur ☐ Tibia ☐ Fibula ☐ Humerus ☐ Radius/Ulna ☐ Other: __________
	Type of Osteomyelitis (Cierny–Mader Classification)	☐ Type I ☐ Type II ☐ Type III ☐ Type IV
	Presence of Sinus or Soft Tissue Defect	☐ Yes ☐ No
	Limb Deformity / Shortening	__________ cm
Laboratory Investigations	ESR (mm/hr)	__________
	CRP (mg/L)	__________
	WBC Count	__________ ×10⁹/L
	Blood Culture	☐ Positive ☐ Negative (If positive, organism: __________)
	Pus / Tissue Culture Result	Organism: __________; Sensitivity pattern: __________
Radiological Findings	X-ray Findings	☐ Sequestrum ☐ Involucrum ☐ Cloaca ☐ Bone Defect Size: __________ cm
	MRI Findings (if available)	__________
Surgical and Treatment Details	Date of Surgery	__________
	Type of Procedure	☐ Debridement ☐ Sequestrectomy ☐ Rod Insertion ☐ Exchange Nailing ☐ Other: __________
	Type of Rod Used	☐ Intramedullary Rod ☐ K-nail ☐ Steinmann Pin ☐ Other: __________
	Cement Type	☐ PMMA ☐ Other: __________
	Antibiotics Mixed in Cement	☐ Vancomycin ☐ Gentamicin ☐ Tobramycin ☐ Other: __________
	Cement-Coating Technique	__________
	Additional Fixation Used	☐ Yes ☐ No (specify): __________
	Duration of Hospital Stay	__________ days
Postoperative Management	Systemic Antibiotics Given	☐ Yes ☐ No (If yes, specify type and duration): __________
	Duration of Rod Retention	__________ weeks
	Wound Healing	☐ Primary ☐ Delayed ☐ Graft/Flap Required
	Time to Start Weight Bearing	__________ weeks
Follow-up Data	Follow-up Duration	__________ months
	Clinical Signs of Infection Resolution	☐ Yes ☐ No
	ESR / CRP Normalization	☐ Yes ☐ No
	Radiological Signs of Healing	☐ Yes ☐ No
	Bone Union Achieved	☐ Yes ☐ No (Time to Union: __________ weeks)
	Limb Function Outcome (ASAMI Score or Similar)	☐ Excellent ☐ Good ☐ Fair ☐ Poor
	Any Complications	☐ Reinfection ☐ Rod Breakage ☐ Persistent Sinus ☐ Nonunion ☐ Other: __________
	Reoperation Required	☐ Yes ☐ No (Reason: __________)
Final Outcome	Infection Control Achieved	☐ Yes ☐ No
	Functional Recovery	☐ Full ☐ Partial ☐ Poor
	Patient Satisfaction	☐ Satisfied ☐ Partially satisfied ☐ Not satisfied
